# Self-rated health among people living with HIV in Spain in 2019: a cross-sectional study

**DOI:** 10.1186/s12879-021-05815-3

**Published:** 2021-01-30

**Authors:** Marta Ruiz-Algueró, Victoria Hernando, Henar Marcos, Gonzalo Gutiérrez, Maria Jesus Pérez-Elías, Juan Carlos López-Bernaldo de Quirós, Federico Pulido, Miguel Górgolas, Jesus Sanz, Ines Suarez-García, Maria Teresa Fernandez, Juan Emilio Losa, Jose Luis Pérez, Maria Oliva Ladrero, Miguel Ángel Prieto, Gustavo González, Ana Izquierdo, Luis Javier Viloria, Irene López, Eva Martínez, Daniel Castrillejo, Rosa Aranguren, Maria Antonia Belmonte, I V Aranda-García, Antonio Arraiza, Asuncion Diaz, on behalf of the Hospital Survey Study Group, M. Egido, S. Letona, M. C. Royo, V. Asensi, E. García, J. Lobo, M. A. Meana, M. de Zárraga, P. Abad, M. Alvarez, R. Suárez del Villar, M. G. Jaume Amengual, A. Rey, A. Payeras, M. Riera, L. Vilaplana, E. Rodríguez de Castro, R. Canet, E. Colino, M. A. Cárdenas, J. L. Gómez, J. Gómez, J. F. Lluch, M. C. Fariñas, E. Martinez, M. I. García, H. Portillo, J. R. Barbera, C. Pereda, G. López, M. P. Geijo, F. Cuadra, M. Torralba, J. M. Yzusqui, M. A. Garcinuño, M. Sánchez, P. Cancelo, J. Locutura, J. A. Carro, A. Bahamonde, Y. Morán, J. Sánchez, A. Iglesias, E. M. Ferreira, M. del Valle, C. Hinojosa, P. Bachiller, A. Chocarro, D. Navarro, L. Mitjans, J. Portilla, M. J. Esteban, M. Masiá, A. Belso, J. Llenas, F. Pasquau, J. Uro, V. Chabrera, M. García, M. J. Galindo, M. Salavert, J. M. Querol, T. Labrador, C. Belenguer, J. Argente, M. J. Garcia, M. Fernández, M. N. Nogales, M. Galán, M. Medina, S. Trejo, C. Martín, C. García, I. Montes, J. A. Oteo, F. Pulido, M. Górgolas, A. Cabello, J. C. López Bernaldo de Quirós, J. Sanz, M. J. Pérez, I. Suárez-García, M. T. Fernández, J. L. Pérez, J. E. Losa, A. Fernández, C. Galera, H. Albendín, G. Alonso, D. Piñar, O. J. Martínez, A. Cano, J. Bravo, A. Arrillaga, J. A. Iribarren, J. J. Portu

**Affiliations:** 1 Unidad de vigilancia de VIH, ITS y hepatitis, Centro Nacional de Epidemiología, Instituto de Salud Carlos III, C/ Monforte de Lemos, 5, 28029 Madrid, Spain; 2 Servicio de Vigilancia Epidemiológica y Enfermedades Transmisibles, DG de Salud Pública, Consejería de Sanidad, Valladolid, Castilla y León Spain; 3 Servicio de Epidemiología, DG de Salud Pública, Consejería de Sanidad, Toledo, Castilla-La Mancha Spain; 4 Servicio de Enfermedades Infecciosas, Hospital Universitario Ramón y Cajal, Madrid, Spain; 5 Servicio de Enfermedades Infecciosas, Hospital Universitario Gregorio Marañón, Madrid, Spain; 6 Unidad VIH, Hospital Universitario 12 de Octubre. imas12.UCM, Madrid, Spain; 7 Unidad de Enfermedades Infecciosas y VIH, Fundación Jimenez Díaz, Madrid, Spain; 8 Unidad de Enfermedades Infecciosas, Hospital Universitario de La Princesa, Madrid, Spain; 9 Grupo de enfermedades infecciosas, Servicio de Medicina Interna, Hospital Universitario Infanta Sofía, San Sebastián de los Reyes, Madrid, Spain; 10 FIIB HUIS HHEN, Universidad Europea, Madrid, Spain; 11 Servicio de Medicina Interna, Hospital del Sureste, Arganda del Rey, Madrid, Spain; 12 Unidad de Enfermedades Infecciosas, Hospital Universitario Fundación Alcorcón, Alcorcón, Madrid, Spain; 13 Servicio de Medicina Interna, Hospital Universitario Infanta Cristina, Parla, Madrid, Spain; 14 Coordinación de VIH/sida, Servicio de Promoción de la Salud y Prevención de la Enfermedad, D.G. de Salud Pública, Zaragoza, Aragón Spain; 15 Servicio de Evaluación de la Salud y Programas, DG de Salud Pública, Consejería de Sanidad, Oviedo, Asturias Spain; 16 Oficina de Coordinación VIH de Extremadura, Servicio de Participación Comunitaria en Salud, DG de Salud Pública, Servicio Extremeño de Salud, Mérida, Extremadura Spain; 17 Servicio de Epidemiología y Promoción de la Salud, DG de Salud Pública, Servicio Canario de la Salud, Santa Cruz de Tenerife, Canarias Spain; 18 Sección de Vigilancia Epidemiológica, DG de Salud Pública, Santander, Cantabria Spain; 19 Servicio de Prevención y Epidemiología del Plan sobre sida, Consejería de Sanidad y Consumo, Ceuta, Spain; 20 Sección de Vigilancia Epidemiológica y Control de Enfermedades Transmisibles, DG de Salud Pública y Consumo, Logroño, La Rioja Spain; 21 Servicio de Epidemiología, DG de Sanidad y Consumo, Consejería de Bienestar Social y Sanidad, Melilla, Spain; 22 Coordinación Autonómica de Drogas y de la Estrategia de Sida, DG de Salut Pública i Consum, Conselleria de Salut, Família i Bienestar Social, Palma de Mallorca, Baleares Spain; 23 Servicio de Promoción y Educación para la Salud, Dirección General de Salud Pública y Adicciones, Consejería de Salud, Murcia, Región de Murcia Spain; 24 Servicio Promoción de la Salud y Prevención en la Etapas de la Vida, Dirección General de Salud Pública y Adicciones, Conselleria de Sanitat Universal i Salut Pública, Valencia, Comunidad Valenciana Spain; 25 Programas de Salud, Direccion General, Osakidetza, San Sebastian, País Vasco Spain

**Keywords:** Self-rated health, Health quality of life, Fourth 90, People living with HIV, Spain

## Abstract

**Background:**

HIV infection has become a chronic disease and well-being of people living with HIV (PLHIV) is now of particular concern. The objectives of this paper were to describe self-rated health among PLHIV, on ART and on ART virally suppressed and to analyse its determinants.

**Methods:**

Data were obtained from a second-generation surveillance system based on a cross-sectional one-day survey in public hospitals. Epidemiological and clinical data were collected among HIV-infected inpatients and outpatients receiving HIV-related care the day of the survey in 86 hospitals in 2019. Self-rated health was measured using a question included in the National Health Survey: “In the last 12 months, how would you rate your health status?” an ordinal variable with five categories (very good, good, moderate, bad and very bad). For the analysis, these responses were dichotomized into two categories: 1 = very good/good and 0 = moderate, bad or very bad health status. Factors associated with very good/good self-rated health were estimated using logistic regression.

**Results:**

Of 800 PLHIV, 67.5% perceived their health as very good/good, 68.4% among PLHIV on ART and 71.7% of those virally suppressed. Having university education (adjusted odds ratio (aOR):2.1), being unemployed (aOR:0.3) or retired (aOR:0.2), ever being diagnosed of AIDS (aOR:0.6), comorbidities (aOR:0.3), less than 2 year since HIV diagnosis (aOR:0.3) and not receiving ART (aOR:0.3) were associated with good self-rated health. Moreover, among PLHIV on ART, viral load less than 200 copies (aOR:3.2) were related to better perceived health. Bad adherence was inversely associated with good self-rated health among PLHIV on ART (aOR:0.5) and of those virally suppressed (aOR:0.4).

**Conclusions:**

Nearly seven in 10 PLHIV in Spain considered their health status as very good/good, being higher among virally suppressed PLHIV. Both demographic and clinical determinants affect quality of life.

## Background

Highly effective antiretroviral treatment (ART) has dramatically changed the natural history of HIV infection in terms of decreasing mortality and increasing life expectancy, thus the HIV infection has become a chronic disease [1]. Benefits of ART go beyond the individual level, since suppressing viral load substantially reduces the risk of HIV transmission [2].

In 2014, the Joint United Nations Programme on HIV/AIDS (UNAIDS) launched the 90–90-90 strategy. This target directed efforts towards testing and treatment in order to achieve the goal of 90% of people living with HIV (PLHIV) being diagnosed, 90% of those diagnosed receiving ART and 90% of those receiving ART having viral load suppression, i.e., at least 73% of all PLHIV worldwide being virally suppressed [3]. Two years later, the World Health Organization (WHO) included the 90–90-90 target in their Global Health Sector Strategy for 2016–2021 to end the acquired immunodeficiency syndrome (AIDS) epidemic as a public health threat by 2030, along with other intermediate objectives to be achieved in 2020. Furthermore, the overall goal included ensuring that PLHIV had healthy lives and promoting well-being for all at all ages [4]. In the same year, Lazarus et al. proposed the concept of the “fourth 90”, with the objective of operationalizing the WHO goal of promoting well-being. The fourth 90 set the objective that 90% of PLHIV with viral load suppression have a good health-related quality of life (HRQoL) [5].

There is no consensus on how to measure HRQoL among PLHIV. Different instruments have been used for this purpose, both generic and HIV-specific scales, that explore different domains [6]. Generic scales have the benefit of allowing data comparisons with the general population [7]; however, they may be less sensitive to changes among the HIV infected population. HIV-specific scales might solve this problem due to their consistency and validity among PLHIV [6]. A recent study in Spain has validated the Spanish version of the WHOQOL-HIV-BREF in a broad sample of HIV-infected people [8]. However, HRQoL measurement with these tools is time-consuming and difficult to incorporate into clinical practice [9].

Self-rated health is a consolidated indicator related to well-being and quality of life [10]. It is considered a predictor of morbidity, mortality and health services use [11, 12] and has been widely used in health and socio-economic surveys at population level in Europe (for instance, in Denmark, Germany, Ireland, Italy, Iceland and Norway) [13, 14].

Several studies have used self-rated health to measure quality of life among PLHIV [15] and PLHIV on ART [16, 17], and have identified related epidemiological and clinical variables. Self-perceived health and comorbidities have been suggested as the two main domains of good HRQoL of PLHIV or “fourth 90” [5]. In the beginning, this new target was described as the last stage of the HIV continuum of care, applied only to PLHIV who were on ART and virally suppressed. However, there is an open debate about whether good HRQoL should be a target only for PLHIV who are virally suppressed or whether it should also cover the previous 90–90-90 and therefore encompass the whole HIV continuum of care [18].

In 2019, between 120,000 and 180,000 people were estimated to be living with HIV in Spain; the estimated HIV prevalence in the age group 15 to 49 years (0.4%) was higher than in other European countries with similar concentrated epidemics, such as France (0.3%) or Italy (0.2%) [19]. At the end of 2019, it was estimated 87% of PLHIV were diagnosed, 97,3% of those diagnosed were receiving ART and 90,4% of those receiving ART having viral load suppression [20]. Recently, several studies have been published exploring HRQoL of PLHIV in Spain, overall and by gender, using HIV-specific scales [8, 21]. However, to our knowledge, there are no studies that analyse self-reported health among PLHIV.

Our aim was to describe self-rated health among PLHIV, among those on ART, and among those on ART that are virally suppressed, and to evaluate factors associated with very good/good self-rated health among these groups in Spain.

## Methods

An observational study was carried out. Data were obtained from a second-generation surveillance system of PLHIV, in Spanish referred to as “Encuesta hospitalaria de pacientes infectados con VIH (EH)” [Hospital survey of patients infected with HIV]. A description of its methodology has been published elsewhere [22]. Briefly, the EH is a cross-sectional survey carried out annually on a specific day. Epidemiological, behavioural and clinical variables were collected among all PLHIV, inpatients and outpatients, attending general public hospitals for HIV-related care on the day of the survey. This population-based information system started in 1996 and over time, variables have been modified to include other information of interest. In the 2019 edition, a question on self-rated health status was introduced. The study was performed in public hospitals. In Spain, HIV care is hospital-based, ART is exclusively provided within hospital pharmacies and HIV-infected subjects living in the catchment area of a public hospital receive care at that hospital. Due to these catchment areas are defined at geographical level and the covered population is known, it is possible to estimate the coverage of the study. ‘Population coverage’ was defined as the total population included in the participating hospitals´ catchment area divided by the total population living in the participating regions. ‘Survey coverage’ was calculated as the total population living in the participating hospitals´ catchment area divided by the total population in Spain.

Participation in the survey is voluntary for both hospitals and individual patients. In 2019 the number of participating hospitals was 86 out of 143 eligible (60.1%) from 15, out of the 19 autonomous regions in Spain. Participating regions were the following (by alphabetical order): Aragón, Asturias, Baleares, Canarias, Cantabria, Castilla-La Mancha, Castilla y León, Ceuta, Comunidad de Madrid, Comunidad Valenciana, Extremadura, Melilla, Murcia, La Rioja and País Vasco (population coverage: 65.5% of the total population in the participating regions). Survey coverage was 38.5% of the whole Spanish population.

Variables were collected from each HIV patient using a standard questionnaire by inpatient and outpatient medical staff. All study variables was extracted from the clinical records, except demographic and self-rated health data, which were obtained directly from the patients by the attending physician. Once completed, the questionnaires were sent to the National Centre of Epidemiology (coordinating center), where questionnaires were entered into a database, made quality control and data analysis.

Variables included in this analysis were the following: a) epidemiological: gender, age, educational level, country of birth, residence, employment status, HIV transmission mode, b) clinical: HIV stage, viral load and CD4 count at last measurement, being on ART, ART adherence, years since HIV diagnosis and comorbidities. ART adherence was classified as optimal, suboptimal or very bad according to the judgment of the attending physician. Comorbidity was gathered as a dichotomous variable that collects the presence of non-AIDS diseases in the previous 12 months (cancer, heart disease, cerebrovascular disease, active hepatitis C, liver cirrhosis, hypertension, mental disorder, kidney disease, respiratory disease).

Self-rated health was measured using the same question included in the last National Health Survey in Spain [23]: “In the last 12 months, how would you rate your health status?” with five response options: very good, good, moderate, bad and very bad. For the analysis, we grouped this variable into two categories: 1 = very good/good and 0 = moderate, bad or very bad health status.

We conducted a descriptive, bivariate and multivariate analysis, according to variables of interest, among all participants, participants on ART and participants on ART virally suppressed. For quantitative variables, median and interquartile range (IQR) were used. For categorical variables, frequency distributions were calculated and the chi-squared test was used to compare percentages. We calculated prevalence of very good/good self-rated health and its 95% confidence interval (95% CI). Logistic regression models were fitted using a backward elimination procedure. Associations were measured using the odds ratio (OR) and its 95% CI. Data analyses were performed using the STATA statistical software package Version 14 (Stata Corp, College Station, Texas, USA).

## Results

In 2019, a total of 843 PLHIV were included in the study. Of these, 800 (94.9%) had data available on self-rated health. Regarding recruitment area, 713 (89.1%) of participants were outpatients, 79 (9.9%) were inpatients and in the remaining 8 subjects (1.0%) this information was unknown. Characteristics of the study population are shown in Table 1. The majority were male, had been born in Spain and were men infected through sexual contact with men (MSM). Median age was 51 years (IQR: 43–56) and 37.4% were between 51 and 60 years old. Nearly 41% had a low educational level (2.5% had no studies and 38.4% only primary education). Regarding residence and employment status, more than half were living with their family and had been employed for the 30 days before the study. Participants had been diagnosed with HIV a median of 14 (IQR: 6–23) years ago. Overall, 776 cases (97.0%) were on ART and of these, 702 (90.5%) had a viral load less than 200 copies/ml at last measurement. Among the 24 cases who were not receiving ART at the time of the study, treatment had been delayed in 15 patients for medical reasons, and the reason was unknown for the remaining cases. The percentage of subjects with a CD4 T-cell count greater than 349 cells was 76.8%. Overall, 11% had other comorbidities and the percentage ever diagnosed with AIDS was 33.4% (267 cases).

**Table 1:** Characteristics of study population and by self-rated health perception, 2019

Variables	Total cases		Self-rated health perception									
			Very good		Good		Moderate		Bad		Very bad	
	n	%	n	%	n	%	n	%	n	%	n	%
Gender												
Male	591	73.9	141	73.1	263	75.8	116	73.4	51	73.9	20	60.6
Female	195	24.4	47	24.4	80	23.1	40	25.3	16	23.2	12	36.4
Transgender	12	1.5	4	2.1	4	1.2	2	1.3	1	1.4	1	3.0
Unknown	2	0.3	1	0.5	0	0.0	0	0.0	1	1.4	0	0.0
Age group (years)												
< 35	111	13.9	48	24.9	38	11.0	17	10.8	5	7.2	3	9.1
35–50	282	35.3	68	35.2	129	37.2	52	32.9	24	34.8	9	27.3
51–60	299	37.4	55	28.5	133	38.3	69	43.7	27	39.1	15	45.5
> 60	95	11.9	16	8.3	44	12.7	17	10.8	13	18.8	5	15.2
Unknown	13	1.6	6	3.1	3	0.9	3	1.9	0	0	1	3.0
Educational level												
Illiteracy	20	2.5	1	0.5	7	2.0	5	3.2	3	4.3	4	12.1
Primary education	307	38.4	49	25.4	132	38.0	72	45.6	39	56.5	15	45.5
Secondary education	258	32.2	74	38.3	109	31.4	52	32.9	15	21.7	8	24.2
University education	180	22.5	62	32.1	88	25.4	22	13.9	7	10.1	1	3.0
Unknown	35	4.4	7	3.6	11	3.2	7	4.4	5	7.2	5	15.2
Country of birth												
Spain	631	78.9	137	71.0	275	79.3	133	84.2	60	87.0	26	78.8
Other	164	20.5	56	29.0	67	19.3	25	15.8	9	13.0	7	21.2
Unknown	5	0.6	0	0	5	1.4	0	0	0	0	0	0.0
Residence												
Living with family	478	59.8	116	60.1	214	61.7	96	60.8	34	49.3	18	54.5
Living alone	216	27.0	55	28.5	87	25.1	42	26.6	24	34.8	8	24.2
Closed institutions	17	2.1	1	0.5	5	1.4	7	4.4	3	4.3	1	3.0
Prisons	9	1.1	1	0.5	4	1.2	1	0.6	3	4.3	0	0.0
Homeless	5	0.6	0	0.0	0	0.0	2	1.3	1	1.4	2	6.1
Other	70	8.8	19	9.8	37	10.7	7	4.4	4	5.8	3	9.1
Unknown	5	0.6	1	0.5	0	0.0	3	1.9	0	0.0	1	3.0
Employment status												
Employed	430	53.8	142	73.6	211	60.8	59	37.3	15	21.7	3	9.1
Unemployed	139	17.4	23	11.9	42	12.1	39	24.7	19	27.5	16	48.5
Retired/disabled	184	23.0	19	9.8	67	19.3	56	35.4	29	42.0	13	39.4
Student	23	2.9	7	3.6	13	3.7	2	1.3	1	1.4	0	0.0
Other/ unknown	24	3.0	2	1.0	14	4.0	2	1.3	5	7.3	1	3.0
Mode of transmission												
Heterosexuals	246	30.8	65	33.7	106	30.5	41	25.9	22	31.9	12	36.4
MSM	293	36.6	83	43.0	145	41.8	44	27.8	17	24.6	4	12.1
PWID	195	24.4	29	15.0	72	20.7	58	36.7	25	36.2	11	33.3
Other/unknown	66	8.3	16	8.3	24	6.9	15	9.5	5	7.3	6	18.2
**Total**	**800**	**100**	**193**	**100**	**347**	**100**	**158**	**100**	**69**	**100**	**33**	**100**

*MSM* Men who have sex with men, *PWID* People who injected drugs

According to self-rated health, 24.1% considered their health as very good, 43.4% good, 19.8% as moderate and 8.6 and 4.1% bad and very bad, respectively.

Prevalence of very good or good self-rated health according to the epidemiological and clinical characteristics is presented in Table 2. Among all patients, this percentage was 67.5%. Prevalence of very good or good self-rated health increased with educational level, and was higher in cases born in countries other than Spain (75.0%) and employed patients (82.1%). Conversely, prevalence of very good/good self-rated health was lower among older age groups and among people who had ever injected drugs (PWID/Ex-PWID) (51.8%), people who lived in closed institutions, prisons or were homeless (35.5%), were unemployed or retired/disabled (47%), and among those diagnosed more than 20 years ago (60.2%). Regarding clinical variables, prevalence of very good or good self-rated health was lower among those ever diagnosed of AIDS (53.9%), patients with viral load more than 200 copies (33.8%) or low CD4 count (20.9%), with comorbidities (33.0%) and not receiving ART (44.4%). All analyzed variables, except gender, were significantly associated with health status in the bivariate analysis (*p* < 0.05).

**Table 2:** Prevalence of very good/good self-rated health among all PLHIV, PLHIV on antiretroviral treatment and PLHIV on antiretroviral treatment virally suppressed

	All PLHIV	Very good/good self-rated health among all PLHIV		On ART	Very good/good self-rated health among PLHIV on ART		On ART virally suppressed	Very good/good self-rated health among PLHIV on ART virally suppressed	
	n	n	% (95% CI)	n	n	% (95% CI)	n	n	% (95% CI)
Gender									
Male	591	404	68.4 (64.4–72.1)	571	398	69.7 (65.8–73.4)	516	377	73.1 (69.0–76.8)
Female	195	127	65.1 (58.0–71.8)	193	126	65.3 (58.1–72.0)	177	120	67.8 (60.4–74.6)
Transgender	12	8	66.7 (34.9–90.0)	11	7	63.6 (30.8–89.1)	8	6	75.0 (34.9–96.8)
Unknown	2	1	50.0 (1.3–98.7)	1	0	0	1	0	0
Age group (years)									
< 35	111	86	77.5 (68.6–84.9)	103	82	79.6 (70.5–86.9)	91	73	80.2 (70.6–87.8)
35–50	282	197	69.9 (64.1–75.2)	274	194	70.8 (65.0–76.1)	240	182	75.8 (69.9–81.1)
51–60	299	188	62.9 (57.1–68.4)	295	187	63.4 (57.6–68.9)	274	182	66.4 (60.5–72.0)
> 60	95	60	63.2 (52.6–72.8)	92	60	65.2 (54.6–74.9)	87	59	67.8 (56.9–77.4)
Unknown	13	9	69.2 (38.6–90.9)	12	8	66.7 (34.9–90.1)	10	7	70.0 (34.8–93.3)
Educational level									
Illiteracy/Primary education	327	189	57.8 (52.2–63.2)	319	188	58.9 (53.3–64.4)	285	176	61.8 (55.8–67.4)
Secondary education	258	183	70.9 (65.0–76.4)	252	180	71.4 (65.4–76.9)	229	172	75.1 (69.0–80.6)
University education	180	150	83.3 (77.1–88.5)	171	146	85.4 (79.2–90.3)	159	139	87.4 (81.2–92.1)
Unknown	35	18	51.4 (34.0–68.6)	34	17	50.0 (32.4–67.6)	29	16	55.2 (35.7–73.6)
Country of birth									
Spain	631	412	65.3 (61.4–69.0)	613	406	66.2 (62.3–70.0)	566	390	68.9 (64.9–72.7)
Other	164	123	75.0 (67.7–81.4)	158	120	76.0 (68.5–82.4)	132	109	82.6 (75.0–88.6)
Unknown	5	5	100.0 (−)	5	5	100 (48.0–100)	4	4	100 (39.8–100)
Residence									
Living with family	478	330	69.0 (63.4–71.9)	467	326	69.8 (65.4–73.9)	428	309	72.2 (67.7–76.4)
Living alone	216	142	65.7 (59.0–72.0)	208	139	66.8 (60.0–73.2)	188	136	72.3 (65.4–78.6)
Closed institutions/Prison/ Homeless	31	11	35.5 (19.2–54.6)	31	11	35.5 (19.2–54.6)	25	9	36.0 (18.0–57.5)
Other	70	56	80.0 (68.7–88.6)	65	54	83.1 (71.7–91.2)	59	48	81.4 (69.1–90.3)
Unknown	5	1	20.0 (0.5–71.6)	5	1	20.0 (0.5–71.6)	2	1	50.0 (1.2–98.7)
Employment status									
Employed	430	353	82.1 (78.1–85.6)	421	348	82.7 (78.7–86.2)	389	333	85.6 (81.7–88.9)
Unemployed	139	65	46.8 (38.3–55.4)	126	61	48.4 (39.4–57.5)	105	54	51.4 (41.5–61.3)
Retired/disabled	184	86	46.7 (39.4–54.2)	182	86	47.2 (39.8–54.8)	167	82	49.1 (41.3–56.9)
Student	23	20	87.0 (66.4–97.2)	23	20	87.0 (66.4–97.2)	22	19	86.4 (65.1–97.1)
Other/ unknown	24	16	66.7 (44.7–84.4)	24	16	66.7 (44.7–84.4)	19	15	78.9 (54.4–93.9)
Mode of transmission									
Heterosexual	246	171	69.5 (63.3–75.2)	240	168	70.0 (63.8–75.7)	216	161	74.5 (68.2–80.2)
MSM	293	228	77.8 (72.6–82.4)	282	223	79.1 (73.9–83.7)	260	210	80.8 (75.4–85.4)
PWID	195	101	51.8 (44.5–59.0)	192	101	52.6 (45.3–59.8)	169	95	56.2 (48.4–63.8)
Other/ unknown	66	40	60.6 (47.8–72.4)	62	39	62.9 (49.7–74.8)	57	37	64.9 (51.1–77.1)
HIV infection stage									
Asymptomatic	386	299	77.5 (73.0–81.5)	378	296	78.3 (73.8–82.4)	353	280	79.3 (74.7–83.4)
Symptomatic non AIDS	126	80	63.5 (54.4–71.9)	122	77	63.1 (53.9–71.7)	111	72	64.9 (55.2–73.7)
AIDS	267	144	53.9 (47.8–60.0)	257	143	55.6 (49.3–61.8)	224	141	63.0 (56.3–69.3)
Unknown	21	17	80.9 (58.1–94.6)	19	15	78.9 (54.4–93.9)	14	10	71.4 (41.9–91.6)
Viral load < 200 copies/ml (last determination)									
Yes	704	503	71.4 (68.0–74.8)	702	503	71.6 (68.2–75.0)	–	–	–
No	71	24	33.8 (23.0–46.0)	56	17	30.4 (18.8–44.0)	–	–	–
Unknown	25	13	52.0 (31.3–72.2)	18	11	61.1 (35.7–82.7)	–	–	–
CD4 count (last determination)									
< 200	67	14	20.9 (11.9–32.6)	61	12	19.7 (10.6–31.8)	36	10	27.8 (14.2–45.2)
200–349	92	48	52.2 (41.5–62.7)	89	47	52.8 (51.9–63.5)	76	91	59.2 (47.3–70.4)
350–499	116	71	61.2 (51.7–70.1)	112	68	60.7 (51.0–69.8)	99	60	60.6 (50.2–70.3)
> =500	499	391	78.4 (74.5–81.9)	496	391	78.8 (75.0–82.3)	483	381	78.9 (75.0–82.4)
Unknown	26	16	61.5 (40.6–79.8)	18	13	72.2 (46.5–90.3)	8	7	87.5 (47.3–99.7)
Comorbidities									
Yes	88	29	33.0 (23.3–43.8)	87	29	33.3 (23.6–44.3)	72	27	37.5 (26.4–49.7)
No/ Unknown	712	511	71.8 (66.3–75.0)	689	502	72.9 (69.3–76.1)	630	476	75.6 (72.0–78.9)
Antiretroviral treatment									
Yes	776	531	68.4 (65.0–71.7)	–	–	–	–	–	–
No	18	8	44.4 (21.5–69.2)	–	–	–	–	–	–
Unknown	6	1	16.7 (0.4–64.1)	–	–	–	–	–	–
Adherence									
Optimal	653	471	72.1 (68.5–75.5)	653	471	72.1 (68.5–75.5)	611	451	73.8 (70.1–77.3)
Suboptimal	68	36	52.9 (40.4–65.1)	68	36	52.9 (40.4–65.2)	56	31	55.4 (41.5–68.7)
Very bad	25	3	12.0 (2.5–31.2)	25	3	12.0 (2.5–31.2)	8	1	12.5 (3.2–52.7)
Unknown	30	21	70.0 (50.6–85.2)	30	21	70.0 (50.6–85.3)	27	20	74.1 (53.7–88.9)
No/unknown ART	24	9	37.5 (18.8–59.4)	–	–	–	–	–	–
Years since HIV diagnosis									
< 2	73	42	57.5 (45.4–69.0)	59	34	57.6 (44.1–70.4)	38	27	71.1 (54.1–84.6)
2–5	111	86	77.5 (68.6–84.8)	109	86	78.9 (70.0–86.1)	99	81	81.8 (72.3–88.6)
6–10	121	93	76.9 (68.3–84.0)	120	93	77.5 (69.0–84.6)	110	89	80.9 (72.3–87.8)
11–15	111	77	69.4 (59.9–77.8)	110	77	70.0 (60.5–78.4)	104	75	72.1 (62.5–80.5)
> 15	374	233	62.3 (57.2–67.2)	369	233	63.1 (58.0–68.1)	345	225	65.2 (60.0–70.2)
Unknown	10	9	90.0 (55.5–99.7)	9	8	88.9 (51.7–99.7)	6	6	100 (54.1–100)
**Total**	**800**	**540**	**67.5** (**64.1–70.7)**	**776**	**531**	**68.4 (65.0–71.7)**	**702**	**503**	**71.7 (68.2–75.0)**

*95% CI* Confidence interval 95%, *PLHIV* People living with HIV, *ART* antiretroviral treatment, *MSM* Men who have sex with men, *PWID* People who injected drugs

Among subjects on ART and those who were on ART and virally suppressed, prevalence of very good or good self-rated health was 68.4 and 71.7%, respectively. Differences in prevalence by variables of interest between these two groups were similar to overall cases. Additionally, this prevalence was higher in patients with optimal ART adherence (Table 2).

In the multivariate analysis, three regression logistic models, adjusted by gender, age, country of birth and transmission mode, were fitted (Table 3). Among PLHIV, having university education was positively associated with having very good or good self-rated health. Factors associated with a poor evaluation of their health status were being unemployed or retired, living in closed institutions/prison/being homeless, ever having been diagnosed of AIDS, having comorbidities, not being on ART and having been diagnosed with HIV less than 2 years ago. Among people on ART, determinants related to reporting better health were similar to those found among overall PLHIV. Moreover, having a viral load less than 200 copies/ml was also associated with good/very good self-rated health. Finally, among cases on ART and those on ART and virally suppressed, suboptimal/very bad adherence to ART were associated with poor self-rated health.

**Table 3:** Factors associated with very good/good self-rated health among all PLHIV, PLHIV on antiretroviral treatment and PLHIV on antiretroviral treatment virally suppressed, 2019

	All PLHIV			PLHIV on ART			PLHIV on ART virally suppressed		
	aOR	95% CI	p	aOR	95% CI	p	aOR	95% CI	p
Gender (male)									
Female	0.7	0.4–1.1	0.164	0.7	0.4–1.1	0.105	0.6	0.4–1.0	0.046
Transgender	1.1	0.2–5.5	0.882	0.9	0.1–5.5	0.899	1.4	0.2–10.5	0.744
Age group (< 35)									
35–50	0.8	0.4–1.5	0.409	0.8	0.4–1.6	0.500	1.1	0.5–2.5	0.737
51–60	0.8	0.4–1.6	0.516	0.7	0.3–1.5	0.344	1.1	0.5–2.3	0.901
> 60	1.6	0.7–3.6	0.298	1.4	0.6–3.5	0.459	2.0	0.8–5.3	0.142
Educational level (Illiteracy/Primary education)									
Secondary education	1.1	0.8–1.7	0.509	1.1	0.7–1.7	0.587	1.2	0.8–2.0	0.382
University education	2.1	1.2–3.8	0.010	2.0	1.1–3.7	0.024	2.1	1.1–3.9	0.028
Country of birth (Spain)									
Other	1.4	0.9–2.3	0.180	1.5	0.9–2.5	0.138	1.6	0.9–3.0	0.106
Residence (living with family)									
Living alone	0.7	0.5–1.1	0.133	0.7	0.5–1.1	0.166	0.8	0.5–1.3	0.450
Closed institutions/Prison/Homeless	0.4	0.1–1.0	0.040	0.4	0.2–1.1	0.088	0.3	0.1–0.9	0.039
Other	1.2	0.6–2.5	0.664	1.3	0.6–2.8	0.575	1.1	0.5–2.4	0.868
Employment status (Employed)									
Unemployed	0.3	0.2–0.4	< 0.001	0.3	0.2–0.5	< 0.001	0.2	0.1–0.4	< 0.001
Retired/disabled	0.2	0.1–0.4	< 0.001	0.2	0.1–0.4	< 0.001	0.2	0.1–0.3	< 0.001
Student	2.0	0.5–7.6	0.313	2.1	0.5–8.6	0.290	2.3	0.6–9.2	0.256
Mode of transmission (heterosexual)									
MSM	1.0	0.6–1.7	0.892	0.8	0.5–1.5	0.507	0.7	0.4–1.3	0.248
PWID	0.7	0.4–1.2	0.174	0.7	0.4–1.2	0.206	0.7	0.3–1.1	0.132
Stage (asymptomatic)									
Symptomatic non AIDS	0.7	0.4–1.2	0.254	0.7	0.4–1.2	0.156	0.6	0.4–1.1	0.128
AIDS	0.6	0.4–0.8	0.006	0.6	0.4–0.9	0.023	0.7	0.5–1.2	0.207
Comorbidities (No/Unknown)									
Yes	0.3	0.2–0.6	< 0.001	0.3	0.2–0.6	< 0.001	0.4	0.2–0.7	0.002
Years since HIV diagnosis (> 15)									
< 2	0.3	0.1–0.6	< 0.001	0.3	0.1–0.6	0.001	0.3	0.1–0.8	0.021
2–5	0.6	0.3–1.1	0.126	0.7	0.3–1.4	0.270	0.8	0.4–1.6	0.519
6–10	0.6	0.4–1.2	0.165	0.7	0.4–1.3	0.264	0.7	0.4–1.4	0.365
11–15	0.6	0.4–1.1	0.118	0.6	0.4–1.1	0.133	0.7	0.4–1.3	0.240
on ART (Yes)									
No	0.3	0.1–0.9	0.036	–	–	–	–	–	–
Viral load < 200 copies/ml (No)									
Yes	–	–	–	3.2	1.5–6.8	0.002	–	–	–
Adherence (optimal)									
Suboptimal/ Very bad	–	–	–	0.5	0.3–0.8	0.006	0.4	0.2–0.8	0.009

*aOR* Adjusted odds ratio, *95% CI* Confidence interval 95%, *PLHIV* People living with HIV, *ART* antiretroviral treatment, *MSM* Men who have sex with men, *PWID* People who injected drugs, Reference categories are in brackets

## Discussion

This manuscript presented data on self-rated health among PLHIV in Spain. Our findings showed the impact of sociodemographic and clinical factors on perceived health. To our knowledge, this is the first study in Spain that provides population-based information on self-rated health using a second generation HIV surveillance system as data source.

Overall, 67.5% of PLHIV perceived their health as very good or good in the previous 12 months. This percentage was 68.4 and 71.7% among PLHIV on ART, and on ART and virally suppressed, respectively. These figures were lower than the 74% reported among the general population by the National Health Survey in Spain, 2017. This difference could be partly explained by the fact that the mean age of analysed PLHIV was higher than the general population included in the Spanish National Health Survey (49 years vs. 43 years, respectively); in fact, positive health perceptions decreased with increasing age in the National Health Survey [23].

Our result was lower than reported in the United Kingdom in 2017 (73% of PLHIV reported very good or good self-rated health) [15] and in South Africa in 2012 (74.1%) [24], although was higher than the 63% reported in Sweden [25]. The United Kingdom study also found a lower prevalence of good/very good self-rated health in PLHIV than in the general population in England (76%) [15]. Among PLHIV on ART, we obtained higher figures than described in Brazil in 2009 (66.4%) [26] and in 2011 (65.0%) [17] in this same subgroup. However, different characteristics of HIV epidemics between countries make the comparisons difficult.

Similarly to other studies in Spain [8, 27] and abroad [17, 28], educational level and employment status were strongly associated with self-rated health in PLHIV, in both ART treated and virologically suppressed. Both variables have been considered a proxy of socio-economic status, which has been shown to be associated with self-rated health among the general population in Spain [23]. Lack of financial and educational resources could increase uncertainty under life circumstances, impairing quality of life. In a cross-sectional study among PLHIV in Canada, a great impact of employment status was found on both physical and mental health quality of life and the authors suggested a bidirectional relationship between both variables: a higher quality of life would be necessary to maintain employment and employment might be a benefit of health and well-being [29]. In our study, being retired was also associated with poor self-rated health suggesting that a person’s financial situation was an important determinant [8].

Lower percentage of very good/good self-rated health was reported among PLHIV who lived in closed institutions, or prisons and those who were homeless. Other studies have described associations between homeless or marginally-housed PLHIV and poor access and adherence to ART, as well as poor retention in care, highlighting the vulnerability of this group of people [30]. On the other hand, lack of social support has been linked to poor health status among PLHIV, either as an independent factor or mediated by depression, isolation or marginalization [8, 28, 31, 32].

Regarding disease stage, people who had ever been diagnosed of AIDS rated their health status as poorer. AIDS has been related to worse physical health and lower scores in mental components of HRQoL [17, 28, 32]. Some cases that reached the last stage of HIV infection are long-term survivors who may have experienced the hardest years of the HIV epidemic with less effective treatments or with more side effects. In our study, 51% of total AIDS cases were diagnosed with HIV infection between 1985 and 2000.

Comorbidities are a major determinant of quality of life for PLHIV, both in the physical [33, 34] and psychological domains [31]. We found a lower prevalence of comorbidities than other studies in Spain [35], suggesting that this second-generation surveillance system did not fully capture all the complexity of multi-morbidity in these patients. In spite of this limitation, our results showed that comorbidities had an important impact on self-rated health among PLHIV, even among patients receiving ART and those with viral suppression. As HIV infection become a chronic disease, other comorbidities have emerged and a comprehensive management of these patients should reinforce preventive measures, early detection, and treatment in order to improve their perceived health.

Aging has been associated with worse HQoL [8, 28]. Some studies have reported a greater impact of age on physical than on mental health [32]; other authors have described a lower prevalence of depression and anxiety in older people [31], related to development of resilience and coping strategies [36]. Our results showed a decreased prevalence of good/very good self-rated health with increasing age in the univariate analysis, but it was no longer statistically significant in the multivariate analysis. This suggests that other factors related to aging, such as comorbidities and ever having been diagnosed of AIDS, rather than biological age, could contribute to poor evaluation of health status in older PLHIV.

A longer time with a diagnosed HIV infection has been associated with lower scores of HQoL [8, 36]. In contrast, our results showed that having had an HIV diagnosis less than 2 years ago was associated with worse self-rated health. One possible explanation for this finding lies in the fact that being diagnosed with HIV infection has been considered a stressful life event with psychological consequences such as depressive and anxiety symptoms [37]. Worries about confidentiality, disclosure, discrimination or stigma, and fear of infecting others, have been described as main stressors among newly HIV diagnosed [38]. Interventions for detecting and reducing stress among recently diagnosed PLHIV will contribute to improve self-rated health.

Being on ART has been associated with better perceived health [28], highlighting the benefits of treatment beyond the clinical and immunological level. Among PLHIV on ART, a suppressed viral load has also been linked to good/very good self-rated health; a better virological status has been related to better physical and mental health [8, 32], although other studies have not found this association [34]. There is more consensus on the relationship between adherence to ART and QoL [8, 39–41]. In our study, poor adherence was related to worse self-rated health among PLHIV on ART and those that were virally suppressed. Improving adherence has benefits not only in slowing disease progression and decreasing mortality, but also in increasing the well-being of PLHIV.

This study has some limitations. Firstly, not all regions in Spain have participated in this information system and results cannot be extrapolated to the whole country. Secondly, participation of hospitals was voluntary, although population coverage regarding population in participating regions was high. Thirdly, patients who attended hospitals more regularly or those who were more seriously ill could be overrepresented; under this hypothesis, prevalence of good or very good self-rated health in this study would be underestimated. Fourthly, many different individuals performed data collection, making it difficult to control reproducibility and data quality. To prevent bias, a common questionnaire and standard procedures were developed. Fifthly, self-rated health is a subjective measure and was difficult to compare with general or specific scales to evaluate HQoL. However, self-perceived health is a multidimensional construction that includes not only health problems but also coping and well-being attitudes. Finally, some important variables that affect HQoL such as depression or anxiety were not included in the EH at the time of the study.

On the other hand, our study also has several strengths. It is population-based, allowing all PLHIV who attend HIV care in the catchment areas of the participating centers to be included. In Spain, ART is available only in hospital pharmacy services and therefore the vast majority of HIV-infected patients receive HIV care and treatment in public hospitals. Inclusion criteria collect both new HIV diagnoses and patients diagnosed many years ago, providing an overall picture of PLHIV in Spain. Furthermore, the use of the same question of self-rated health than in the National Health Survey allowed for comparison with the general population. This new variable has been well accepted by participants, as indicated by the low number of missing data. Last but not at least, including a self-rated health question in a consistent information system will allow us to include a proxy of HQoL as part of routine monitoring of PLHIV.

## Conclusions

Nearly seven in 10 participants in this study considered their health status as good or very good; this figure was lower than in the general population in Spain, even among PLHIV who were virally suppressed. Both demographic and clinical determinants had an impact on quality of life.

Prevalence of very good/good self-rated health increased among PLHIV on ART and among those virally suppressed. Measuring this indicator only in the last subgroup does not take into account HIV-infected people who not receiving ART and those on ART with unsuppressed viral load; these two groups perceived having poorer health. This finding suggested that evaluating self-rated health as a proxy of the fourth 90 only among virally suppressed PLVIH could provide overestimated results.

## Data Availability

The dataset analysed during the current study is only available from the corresponding author on reasonable request.

## Abbreviations

AIDS: Acquired immunodeficiency syndrome
ART: Antiretroviral treatment
HRQoL: Health-related quality of life
MSM: Men who have sex with men
PLHIV: People living with HIV
PWID/Ex-PWID: People who had ever injected drugs
